# Derivation of a clinical decision-making aid to improve the insertion of clinically indicated peripheral intravenous catheters and promote vessel health preservation. An observational study

**DOI:** 10.1371/journal.pone.0213923

**Published:** 2019-03-22

**Authors:** Peter J. Carr, James C. R. Rippey, Marie L. Cooke, Niall S. Higgins, Michelle L. Trevenen, Aileen Foale, Gerben Keijzers, Claire M. Rickard

**Affiliations:** 1 Health Research Board, Clinical Research Facility, National University of Ireland Galway, Galway, Ireland; 2 Alliance for Vascular Access Teaching & Research (AVATAR) Group, Menzies Health Institute Queensland, School of Nursing and Midwifery, Griffith University, Brisbane, Australia; 3 School of Medicine, Faculty of Health and Medical Sciences, The University of Western Australia, Nedlands, Perth, Australia; 4 Sir Charles Gairdner Hospital, QEII Medical Centre, Nedlands, Perth, Western Australia; 5 Queensland University of Technology, Brisbane, Australia; 6 Centre for Applied Statistics, The University of Western Australia, Nedlands, Australia; 7 Fiona Stanley Hospital, Murdoch, Australia; 8 Department of Emergency Medicine, Gold Coast University Hospital, Gold Coast; 9 Australia School of Medicine, Bond University, Gold Coast, Australia; 10 School of Medicine, Griffith University, Gold Coast, Australia; University of Wisconsin, UNITED STATES

## Abstract

**Background:**

It is well established that the idle peripheral intravenous catheter (PIVC) provides no therapeutic value and is a clinical, economic and above all, patient concern. This study aimed to develop a decision aid to assist with clinical decision making to promote clinically indicated peripheral intravenous catheter (CIPIVC) insertion in the emergency department (ED) setting. Providing evidence for a uniform process could assist clinicians in a decision-making process for PIVC insertion. This could enable patients receive appropriate vascular access healthcare.

**Methods:**

We performed a secondary analysis of data from a multicentre cohort of emergency department clinicians who performed PIVC insertion. We defined CIPIVC a priori as one used for a specific clinical treatment and or procedure such as prescribed intravenous (IV) fluids; prescribed IV medication; or IV contrast (for computerized tomography scans). We sought to refute or validate an assumption if the clinician performing or requesting the insertion decided the patient was >80% likely to need a PIVC. Using logistic regression, we derived a decision aid for CIPIVCs.

**Results:**

In 817 patients undergoing PIVC insertion, we observed 68% of these to be CIPIVCs. Admitted patients were significantly more likely to have a CIPIVC, Odds Ratio (OR) = 3.05, 95% confidence interval (CI) = 2.17–4.30, p = <0.0001. Before insertion, patients who definitely needed IV fluids/medicines OR = 3.30, 95% CI = 2.02–5.39, p = <0.0001 and who definitely needed a contrast scan OR = 3.04, 95% CI = 1.15–8.03, p = 0.0250 were significantly more likely to have a device inserted for a clinical indication. Patients who presented with an existing vascular access device were more likely to have a new CIPIVC inserted for use OR = 4.35, 95% CI = 1.58–11.95, p = 0.0043. The clinician’s pre-procedural judgment of the likelihood of therapeutic use >80% was independently associated with CIPIVC; OR 3.16, 95% CI = 2.06–4.87, p<0.0001. The area under the receiver operating characteristic curve was 0.81, and at the best cut-off, the model had a specificity of 0.81, sensitivity of 0.71, a positive predictive value of 0.89 and negative predictive value of 0.57.

**Conclusions:**

Using the derived decision aid, clinicians could ask:- “Does this patient need *A-PIVC*?” Clinicians can decide to insert a CIPIVCs when: (i) **A**dmission to hospital is anticipated and when (ii) a **P**rocedure requires a PIVC, e.g., computerised tomography scans and where an existing suitable vascular access device is not present and or; (iii) there is an indication for **IV** fluids and or medicines that cannot be tolerated enterally and are suitable for dilution in peripheral veins; and, (iv) the **C**linician’s perceived likelihood of use is greater than 80%.

## Introduction

The annals of vascular access history show that we have attempted to perform peripheral intravenous cannulation since 1492 [1]. In the centuries since, ethical principles such as beneficence and non-maleficence underpinned by the concepts “first do no harm” or “first do no net harm” have developed to substantiate part of a clinician’s clinical decision making [2]. The peripheral intravenous catheter (PIVC), is the most prevalent of all vascular access devices and is primarily initiated when intravenous fluids and or medication is prescribed, to relieve patients of pain and/or assist patients to better health [3]. In contrast, the insertion of an unnecessary PIVC challenges the concept of first do no harm [4]. Current evidence of unused PIVC rates for patients admitted via the Emergency Department (ED) setting range from 25–50% [5–7]. Additionally, avoiding inappropriate PIVC placement upholds the concept of vessel health and preservation [8].

The revelation that up to half of all PIVCs inserted in the ED are unused is disturbing and has prompted a closer examination of this clinical procedure [5]. Becerra and colleagues performed an integrative review on the prevalence of idle PIVCs and called for stronger criteria for clinically indicated peripheral intravenous catheter (CIPIVC) use [9]. The review demonstrated heterogeneous definitions of the idle PIVC (those not deemed to be a CIPIVC) and varied from: the length of time (hours and days); in situ without infusion therapy; lack of prescribed intravenous fluids or medicines; and even the unspecified descriptor of patient instability [9]. More recently a specific ED review on idle PIVCs identifies a median prevalence of idle PIVC to be 32.4% [10].

At a minimum, refraining from inserting a PIVC that is not clinically indicated would avoid pain, and reduce costs of staff and equipment resources involved. Besides, it would not place the patient at risk of more serious complications such as vein injury and thrombophlebitis. Blood sampling from PIVCs inserted in the ED is very common as it is thought to preserve the patient’s vessel health and thus a repeated needle insertion is avoided. Unfortunately, it is this sort of practice that contributes to an unnecessary insertion when the PIVC is used for one-off blood sampling and not for intravenous fluids and or medication infusions. Thus, the term the idle PIVC is coined. Furthermore, the ED inserted PIVC that remains intravenous without a clinical indication contributes to hospital-acquired infection risk including staphylococcus aureus bloodstream infections [11]. Therefore, given the morbidity and cost associated with catheter-related infections, better clinical decision aids to avoid unnecessary PIVCs in the first place may address clinical and economic concerns.

The process of describing PIVC use is best understood when separated into three distinct phases: pre-insertion; insertion; and post insertion. Focusing on the pre-insertion phase (as this paper does) and developing a uniform set of criteria for CIPIVC insertion, facilitates the reduction of unnecessary PIVC attempts. Proper attention paid to this phase will avoid the insertion of “just in case” PIVCs. The PIVC is an invasive device that comes with a variety of risks and it should be dependent upon a well-defined clinical rationale for insertion to proceed. Patients, must, where possible, be made aware of these risks. This study intended to improve the quality of clinical practice with vascular access in the ED and specifically, to describe factors associated with the clinical indication for a PIVC insertion [12]. Given the fact that PIVC assessment tools have shown questionable clinical reliability and PIVC removal is poorly recorded in the medical record [13,14] better strategies are needed to improve data capture and perhaps identify why PIVCs are clinically justified is a good start.

## Methods

### Study aim

We aimed to identify factors associated with CIPIVC status so that we could develop a clinical decision aid for CIPIVC insertion to facilitate decision-making and reduce unnecessary PIVCs.

### Study design, setting, sampling and participants

We performed secondary data analysis on a dataset regarding of PIVC insertion outcomes in the ED setting [12] which was registered as a clinical trial with the Australian and New Zealand Trials Registry (ANZCTRN12615000588594). The study setting included two large academic affiliated institutions in Perth, Western Australia. The first is a 650-bed hospital treating approximately 65,000 patients present annually in the ED. The other is a 783-bed hospital with approximately 80,000 adult ED presentations [12]. The dataset was developed using a convenience sampling method due to limited funding and included all patients with various Australasian Triage Scale (ATS) 1–5, who received a PIVC.

### Study definitions

For this study and analysis, we defined CIPIVCs that were used for a: (i) clinical procedure requiring intravenous contrast or medicines; (ii) prescribed IV fluids and or IV medication (IVFM) in the ED or during admission to hospital.

### Data collection

We collected data from June 2015 to May 2016 using a case report form that we had developed prior to the main study and was assessed as having an item content validity index score of greater than 0.78, suggesting good content validity [15]. Two research assistants separately gathered observation data on PIVC insertion and followed up those admitted to hospital for use until removal. Follow up data included patient, clinician, and product factors. Before insertion, the inserting clinician was asked how likely the PIVC would be needed for clinical use on a scale of 0% = not likely to be needed, to 100% = extremely likely to be needed.

Additionally, clinicians were asked if the purpose was for blood sampling and if intravenous fluids; medicines; and contrast scan would be definitely or possibly prescribed. We then followed up the patients to see if fluids and or medicines were prescribed and administered. A sample of data from each was assessed initially and obtained high-reliability scores. Kappa was above 0.90 suggesting a very high level of agreement [16].

### Data analysis

Summary statistics are provided for all variables of interest, including means and standard deviations (SD) for continuous variables as well as counts (N) and percentages (%) for each of the categorical variables. Predictors of CIPIVC insertion were identified using univariate and multivariate logistic regression to investigate variables related to whether the PIVC was clinically indicated (event = “PIVC clinically indicated”). Backwards model selection was used where variables significant at the 5% level were retained for the final multivariate model. Adjusted odds ratios (OR), 95% confidence intervals (CI), and P-values are provided. Data were analysed using the R environment for statistical computing [17].

Ethics: Approvals for this study were obtained from The Sir Charles Gairdner Hospital (SCGH) Human Research Ethics office ref: HR 2015–149 with reciprocated approval gained at Fiona Stanley Hospital and Griffith University. We observed only clinicians who, after an invitation to contribute to the study, provided consent. A waiver of consent was granted to allow us observe patients receiving PIVCs. We used the STROBE statement to report our findings.

## Results

Table 1 displays patient characteristics for the 817 patients with observed PIVC insertions including those that were CIPIVCs (n = 553; 67.7%) and those that were not CIPIVCs (n = 264; 32.3%). There were more female patients n = 431 (53%) represented, and the mean age was 60 (SD 22) years. The most common triage category was ATS 3 (n = 339; 42%), followed by ATS 2 (n = 277; 34%). The professional designation of those who performed the most PIVC insertions was the resident medical officer (RMO; n = 343; 42%), with 385 (47%) clinicians having inserted more than 1000 PIVCs in their career. PIVCs were inserted for the initial purpose of obtaining a blood sample in 748 (92%) patients, with 292 (36%) not having any IV fluids, IV medication infused. Four-hundred and five (84%) patients with a pre-insertion likelihood of use of greater than 80%, received CIPIVC, whereas 148 (44%) of patients with a pre-insertion clinician-estimated likelihood of use less than 80% received a CIPIVC.

**Table 1:** Overall patient characteristics and by CIPIVC insertion classification.

	CIPIVC Insertion		Overall (N = 817)
	Yes (N = 553; 67.7%)	No (N = 264; 32.3%)	
**Patient Gender**			
Male	270 (70.0%)	116 (30.0%)	386 (47.3%)
Female	283 (65.7%)	148 (34.3%)	431 (52.7%)
**Patient Age**			
Years (Mean, SD)	59.8 (22.6)	60.8 (22.0)	60.1 (22.4)
**Triage Category**			
1 -	32 (82.1%)	7 (17.9%)	39 (4.8%)
2 -	185 (66.78%)	92 (33.2%)	277 (33.9%)
3 -	236 (69.6%)	103 (30.4%)	339 (41.5%)
4 -	97 (61.8%)	60 (38.2%)	157 (19.2%)
5 -	3 (60.0%)	2 (40.0%)	5 (0.6%)
**Staff Role**			
Nurse	44 (58.7%)	31 (41.3%)	75 (9.2%)
Med Student	29 (69.1%)	13 (30.9%)	42 (5.1%)
Intern	57 (63.3%)	33 (36.7%)	90 (11.0%)
RMO	225 (65.6%)	118 (34.4%)	343 (42.0%)
Registrar	93 (69.4%)	41 (30.6%)	134 (16.4%)
Consultant	44 (80.0%)	11 (20.0%)	55 (6.7%)
US Consultant	12 (92.3%)	1 (7.7%)	13 (1.6%)
Phlebotomist	49 (75.4%)	16 (24.6%)	65 (8.0%)
**Staff Experience**			
<10	3 (50.0%)	3 (50.0%)	6 (0.7%)
11–50	31 (66.0%)	16 (34.0%)	47 (5.8%)
51–100	30 (60.0%)	20 (40.0%)	50 (6.1%)
101–300	71 (69.6%)	31 (30.4%)	102 (12.5%)
301–600	60 (61.9%)	37 (38.1%)	97 (11.9%)
601–1000	85 (65.4%)	45 (34.6%)	130 (15.9%)
>1000	273 (70.9%)	112 (29.1%)	385 (47.1%)
**Patient Admitted**			
Yes	371 (78.4%)	102 (21.6%)	473 (57.9%)
No	182 (52.9%)	162 (47.1%)	344 (42.1%)
**Existing Device**			
Yes	50 (90.9%)	5 (9.1%)	55 (6.7%)
No	503 (66.0%)	259 (34.0%)	762 (93.3%)
**Blood Samples**			
Yes	497 (66.4%)	251 (33.6%)	748 (91.56%)
No	56 (81.2%)	13 (18.8%)	69 (8.4%)
**Possible IVT**			
Yes	152 (54.7%)	126 (45.3%)	278 (34.0%)
No	401 (74.4%)	138 (25.6%)	539 (66.0%)
**Definite IVT**			
Yes	307 (88.5%)	40 (11.5%)	347 (42.5%)
No	246 (52.3%)	224 (47.7%)	470 (57.5%)
**IVT Infused**			
Yes	525 (100%)	0 (0.00%)	525 (64.3%)
No	28 (9.59%)	264 (90.4%)	292 (35.7%)
**Possible Contrast**			
Yes	26 (56.5%)	20 (43.5%)	46 (5.6%)
No	527 (68.4%)	244 (31.6%)	771 (94.4%)
**Definite Contrast**			
Yes	25 (80.7%)	6 (19.35%)	31 (3.8%)
No	528 (67.2%)	258 (32.82%)	786 (96.2%)
**CT Scan**			
No CT Scans	368 (62.6%)	220 (37.4%)	588 (72.0%)
CT Non-Contrast	69 (61.1%)	44 (38.9%)	113 (13.8%)
CT +Contrast	116 (100%)	0 (0.00%)	116 (14.2%)
**Blood Products**			
Yes	9 (64.3%)	5 (35.7%)	14 (1.7%)
No	544 (67.8%)	259 (32.3%)	803 (98.3%)
**Code Black**			
Yes	12 (80.0%)	3 (20.0%)	15 (1.8%)
No	541 (67.5%)	261 (32.5%)	802 (98.2%)
**Patient Unstable**			
Yes	82 (78.1%)	23 (21.9%)	105 (12.9%)
No	471 (66.2%)	241 (33.8%)	712 (87.1%)
**How Likely** ≤80%	148 (44.2%)	187 (55.8%)	335 (41.0%)
>80%	405 (84.0%)	77 (16.0%)	482 (59.0%)
**Hospital**			
SCGH	322 (71.1%)	131 (28.9%)	453 (55.5%)
FSH	231 (63.5%)	133 (36.5%)	364 (44.5%)

Standard deviation SD; computerised tomography CT; Intravenous therapy IVT; Sir Chares Gairdner Hospital SCGH; Fiona Stanley Hospital FSH; Ultrasound US

### Regression results

**Fig 1:**
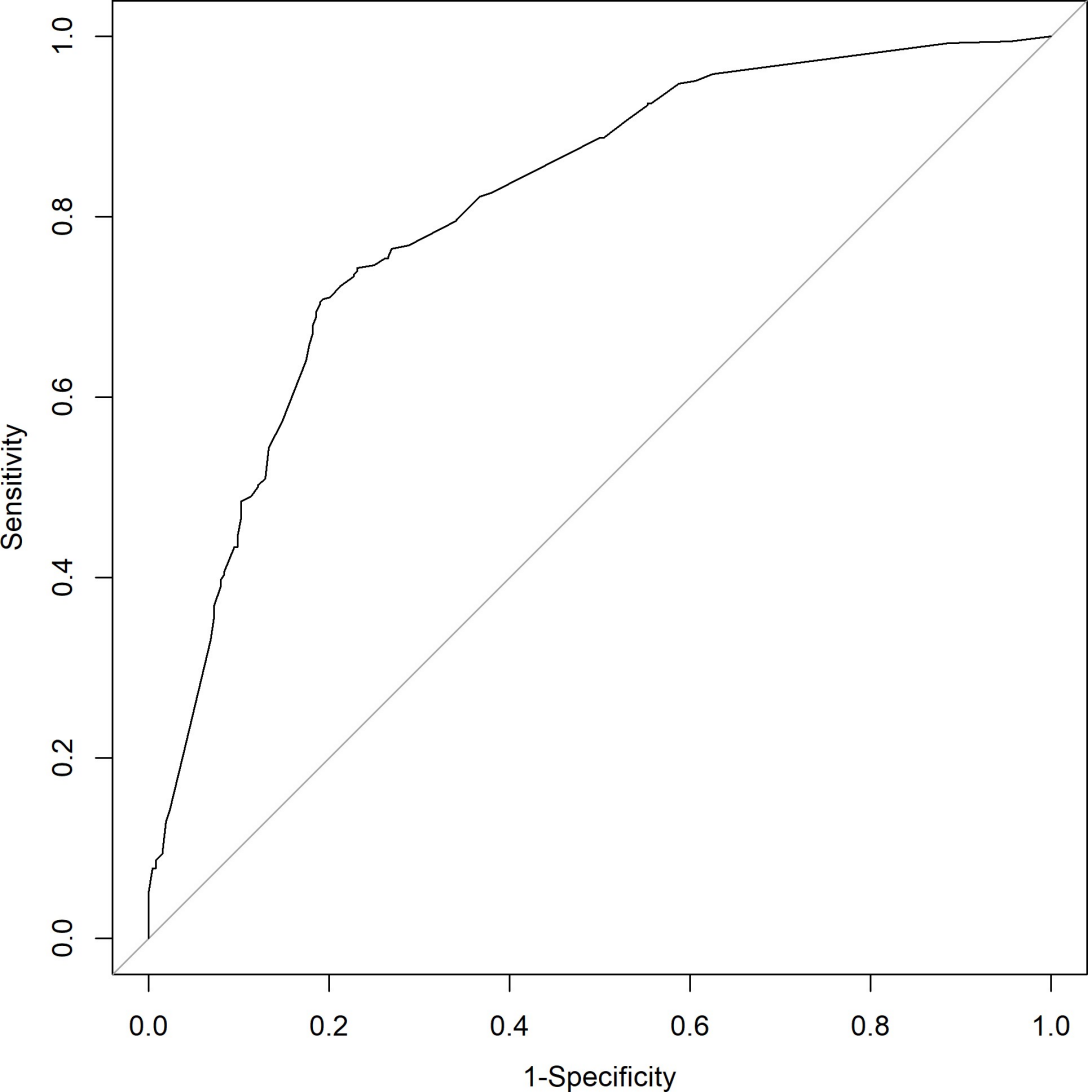
Receiver operator characteristic curve of the final multivariate model.

Table 2 displays the univariate and multivariate results from analyzing whether a PIVC insertion was clinically indicated as per the definition, while Fig 1 displays the receiving operating characteristic curve produced by the final multivariate model. Those with blood sampling obtained were less likely to require CIPIVC insertion (univariate OR = 0.46, 95% CI = 0.25–0.86) but this was not significant in the multivariate analysis. Nurses were the most likely to insert an unused PIVC; however, this relationship did not reach statistical significance. Furthermore, no statistically significant associations between requiring a CIPIVC and patient age, patient gender, and staff experience were observed. The final multivariate model found five independent factors associated with CIPVC insertion; (i) admitted patients were more likely to receive a CIPIVC (OR = 3.05, 95% CI = 2.17–4.30, p = <0.0001); (ii) patients with an existing device were significantly more likely to require a CIPIVC (OR = 4.35, 95% CI = 1.58–11.95, p = 0.0043); (iii) patients predicted by clinicians as definitely needing IVT prior to insertion were significantly more likely to require a CIPIVC than patients who were not (OR = 3.30, 95% CI = 2.02–5.39 p = <0.0001); (iv) patients predicted to definitely need a contrast scan were significantly more likely to require a CIPIVC (OR = 3.04, 95% CI = 1.15–8.03, p = 0.0250); and (v) patients predicted pre-procedurally to have >80% likelihood of use were significantly more likely to require a CIPIVC (OR 3.16, 95% CI = 2.06–4.87, p<0.0001).

**Table 2:** Univariate and multivariate logistic regression of whether the PIVC was used or not.

	UNIVARIATE		MULTIVARIATE		
	OR	95% CI	OR	95% CI	P-value
**Patient Gender**					
Female vs. Male	0.82	0.61–1.10	Not Significant		
**Triage Category**					
1 vs. 5	3.05	0.43–21.79	Not Significant		
2 vs. 5	1.34	0.22–8.16			
3 vs. 5	1.53	0.25–9.28			
4 vs. 5	1.08	0.18–6.64			
**Staff Role**					
*Consultant vs. Nurse	3.29	1.52–7.13	Not Significant		
Intern vs. Nurse	1.22	0.65–2.28			
Med Student vs. Nurse	1.57	0.71–3.50			
Phlebotomist vs. Nurse	2.16	1.04–4.47			
RMO vs. Nurse	1.34	0.81–2.24			
Registrar vs. Nurse	1.60	0.89–2.88			
**Staff Experience**					
301–1000 vs. <301	0.92	0.62–1.36	Not Significant		
>1000 vs. <301	1.26	0.88–1.82			
**Patient Admitted**					
Yes vs. No	3.24	2.39–4.39	3.05	2.17–4.30	<0.0001
**Existing Device**					
Yes vs. No	5.15	2.03–13.07	4.35	1.58–11.95	0.0043
**Blood Samples**					
Yes vs. No	0.46	0.25–0.86	Not Significant		
**Possible IVT predicted**					
Yes vs. No	0.42	0.31–0.56	Not Significant		
**Definite IVT predicted**					
Yes vs. No	6.99	4.80–10.18	3.30	2.02–5.39	<0.0001
**Possible Contrast predicted**					
Yes vs. No	0.60	0.33–1.10	Not Significant		
**Definite Contrast predicted**					
Yes vs. No	2.04	0.83–5.03	3.04	1.15–8.03	0.0250
**Blood Products**					
Yes vs. No	0.86	0.28–2.58	Not Significant		
**Code Black**					
Yes vs. No	1.93	0.54–6.90	Not Significant		
**Patient Age**					
For a One Year Increase	1.00	0.99–1.01	Not Significant		
**How Likely**					
>80% vs ≤80%	6.65	4.80–9.20	3.16	2.06–4.87	<0.0001
**Deterioration/Patient Unstable**					
Yes vs. No	1.82	1.12–2.97	Not Significant		
**Hospital**					
FSH vs. SCGH	0.71	0.53–0.95	Not Significant		

*Combined consultants and ultrasound accredited consultants.

Standard deviation SD; computerised tomography CT; Intravenous therapy IVT; Sir Chares Gairdner Hospital SCGH; Fiona Stanley Hospital FSH, RMO Resident medical officer

Fig 1 displays the area under the receiver operating characteristic curve of the final multivariate model which was 0.81, and at the best cut-off the model yielded a specificity of 0.81, sensitivity of 0.71 a positive predictive value of 0.89 and negative predictive value of 0.57.

## Discussion

Our study shows that one-third of patients in two large Emergency Departments receive a PIVC that is not clinically indicated based on our definition. This finding should raise both clinical and economic concerns. We have identified five factors that are associated with CIPIVC insertion and these can be used to guide decision making about whether to insert a PIVC in the first place.

Admission to hospital was independently associated with CIPIVC inserted in ED. This describes good practice for those that require it. However, the indiscriminate practice of PIVC without a clinical indication is detrimental to good clinical care. If the clinician considers discharge highly likely, the question of clinical indication for PIVC becomes even more pertinent. We identified that over 22% of the admitted cohort had a PIVC inserted with no intravenous therapy, medication infused, and/or no CT contrast scan performed. Despite almost one in three of all PIVCs having no clear indication or justification, it is unclear if these PIVCs were all unjustified. At the time of insertion in ED, the clinician is likely focused on obtaining PIVC for blood sampling and being cognizant of potential patient deterioration in an undifferentiated patient and therefore, influencing the perception of what constitutes CIPIVC.

Additionally, the presence of an existing pre-hospital PIVC or patients with an existing vascular access device (VAD) did not negate PIVC insertion, and this was statistically significant. This emphasizes clinicians continue to rely on the PIVC as a reliable device to use despite post insertion complications and high failure rates [18,19]. It is worthy of further investigation as to why clinicians feel the need to instigate another PIVC rather than critically assess the utility of the pre-hospital PIVC initiated elsewhere. Furthermore, as to why other existing devices such as centrally terminating catheters were not used demands more investigation. Not all ED clinicians are familiar with using all vascular access devices. Regarding preserving vessel health, perhaps the first consideration where possible and appropriate could be given to using the existing device. Conversely, clinicians may feel ill-trained to manage such devices, are concerns in case infection occurs or perhaps lack the necessary skills to do so, this is an issue certainly worthy of further inquiry. Furthermore, at a minimum and where practical, patient input should be included given the growing appreciation of shared decision-making concept.

Significantly, prior to the insertion of a PIVC clinicians were able to predict the utility of the device in terms of whether definitive intravenous therapy would be infused. In future it may be worth assessing if any of these infusates could convert to an enteral prescription to avoid PIVC insertion. Conversely, however, if the infusate is inappropriate for enteral prescription but is likely to cause premature PIVC failure then selecting an appropriate device may advantage both the patient and service provider [20]. Such a scenario would be dependent on a system to resource point of care vascular access provision. Additionally, if definitive contrast CT scanning was predicted this was significantly related to a CIPIVC.

The final independent predictor in our analysis assesses the complexity of indications for PIVC insertion, and we adopted a simple cut off percentage point of 80% for the anticipated therapeutic use proposed by Kelly and Egerton-Warburton [21]. Encouragingly, this was recently validated in a pre and post implementation study adopting *are you 80% sure*? as an important predictor for a CIPIVC [22]. Our decision aid derived from a logistic regression method suggests that clinician gestalt [23] represented by the clinician ability to predict PIVC utility to be >80% represented an appropriate percentage for CIPIVC, thus providing further evidence to support the concept of *are you 80% sure*? [22]. This is important given the call by Becerra and colleagues for a uniform definition for the term ‘idle PIVC’ and as such is telling of a system failure to understand or adequately rationalise any plausible clinical rationale for patients receiving a PIVC [9].

No relationship was detected between the Australasian Triage Scale (ATS) and whether the PIVC was clinically indicated in our multivariate analysis. However, this must be interpreted cautiously as some ATS 1 (immediately life-threatening) and 2 (imminently life-threatening) may have additional PIVCs inserted, as the anticipation of PIVC use is greatest in the ATS 1 and 2. For example, the ATS 1 with unused PIVCs in our dataset included patients with the following presenting complaints and diagnoses which are at risk of acute deterioration: acute stroke; acute shortness of breath with chronic airways disease; chest pain; arterial laceration; code black/social presentation. All of which are likely contributing factors to a wide confidence interval (CI) evidenced in the univariate analysis in Table 2.

There was a trend toward nurses being the professional group most likely to insert unused PIVCs; however, it is not known if these were nurse-initiated decisions or a consequence of a medical order or other (perceived) workflow processes. This is not a unique interpretation of data regarding unused PIVCs as other reports revealing 50% rate of idle PIVCs identified that nursing staff inserted 80% of the PIVCs [5]. This report also used a similar definition to our CIPIVC. It accentuates the need for nurses to take a more involved role in decision-making and as such could be improved with a decision aid. That said, more scientific and health service enquiry should be carried out to identify why any clinical staff inserts PIVCs that are not deemed clinically indicated.

### *A-PIVC* decision aid

We present the *A-PIVC* decision aid which includes the following: admission to hospital;, with the following circumstances; procedures requiring a PIVC e.g. computerised tomography scans and where an existing device is not present; and or; indication for IV fluids and or medicines that cannot be tolerated enterally and are suitable to for dilution in peripheral veins; clinician likelihood of therapeutic use is greater than 80%, see a fuller explanation in S1 and S2 Figs. Although admitted patients were independently associated with CIPIVC, it is worth emphasizing that CIPIVC can occur in the ED patient who does not get admitted. Additionally, a PIVC might be the inappropriate vascular access device choice, and alternative devices could be considered for admitted patients. Furthermore, patients sometimes are admitted for supportive care and allied health input and do not require imaging or IV fluids and medications. This subgroup of patients (at low risk of deterioration) could be admitted without PIVC. Therefore, we suggest an admitted cohort receive a CIPIVC when other indications will occur as outlined in Table 3.

**Table 3:** The *A-PIVC* decision aid to support CIPIVC use.

**A**	**A**dmission to hospital with the any of the following below
**P**	**P**rocedures requiring a PIVC (CT contrast scan); **Check for the** p**resence of an existing device but consider whether this could be used rather than inserting a new PIVC.**
**I** **V**	**I**v Fluids indicated where an equivalent cannot be tolerated enterally. i**V** Medicines that cannot be tolerated enterally and are suitable for dilution in peripheral veins.
**C**	**C**linician predicts likelihood of use is over 80%.

We expect our pragmatic *A-PIVC* decision aid will likely reduce the unwarranted idle PIVC and likely support resource stewardship initiatives and perhaps facilitate appropriate vascular access device placement. *A-PIVC* could better assist decisions for all ED staff to insert CIPIVCs. The checking for the presence of an existing device or the use of alternative devices for infusates that are likely to fail when infused via a PIVC can improve patient outcomes. As a result it implements the concept of vessel health and preservation philosophy of the right device for the right infusates at the right time, a valuable clinical notion [8,24–26]. This decision aid may appropriately decrease incidence of idle PIVCs and as a post insertion strategy as it has the potential to guide decisions to remove PIVCs where no clinical indication exists.

Additionally, where the PIVC is the inappropriate VAD and therefore contributing to infusion harm compromising vessel health and preservation, it suggests considering an alternative VAD. However, decision tools or aids assisting with the identification of the appropriate VAD selection and placement will require educational investment and resource planning. Such a concept may yield greater outcomes for those admitted [8,20,27] and conceivably with a vascular access specialist team leading such an initiative [28].

The proportion of PIVCs inserted not associated with clinical utility such as a computerised tomography contrast scan; evidence of intravenous fluids or medicines transfused; was 32%. However, and perhaps, encouraging, is that our proportion of idle PIVCs is much less than that reported by Limm and colleagues in another Australian ED (50%), [5] but similar to a single centre retrospective ED study regarding the clinical utility of the PIVC [29].

## Limitations

There are a number of limitations to our findings. Firstly, our sample is convenient and not consecutive. Secondly, we could not accurately report if the PIVC was used for serial blood sampling (particularly important in cardiac cases); additionally, we did not assess patient choice/shared decision-making when PIVCs were inserted. Thirdly, when an ED PIVC was inserted in someone with a pre-hospital PIVC we could not accurately identify which one was used and if any were used for blood sampling, but results suggest preservation of veins or reluctance to use an existing device is worthy of further exploration. Fourth, our data assessed for all factors related to the device being used but we did not monitor if the patient was likely to clinically deteriorate, and therefore some of the PIVCs may be justified as per local policy. We did not assess what pre-registration; post-registration or what continuing education clinicians possessed or are available and whether this was associated with a CIPIVC insertion. Finally, while having IV fluid or IV medication could be seen as clinically appropriate, we highlight a limitation of reverse causation–meaning that fluid or medication was administered because the patient had a PIVC or given IV antibiotics where oral may have sufficed. This could have led to an underestimation of inappropriate PIVCs.

## Conclusions

Prior to insertion a PIVC must be clinically indicated appropriate for the duration of patient care. On the basis of our data and interpretation noting that external validation is required, ED clinicians should simply ask does the patient they are caring for need *A-PIVC* by using our novel decision aid. We hope *A-PIVC* will support a standard measurement for PIVC procedures and when guided by local policy, driven by a clinical decision that, where possible, includes the patient in the decision-making process. To our knowledge, this is one of the first reports to develop a decision aid for a clinically indicated PIVC using a large prospective dataset with a logistic regression technique. Future planned evaluation and validation studies of *A-PIVC* are necessary and can address the limitations identified, but we believe that this supports other global *Choosing Wisely* initiatives on this topic. Finally, the *A-PIVC* aid could support purposeful clinician inertia on this topic. In the centuries since we have performed this procedure it is past time that a decision regarding the insertion of a needle and plastic tube in a person’s venous anatomy is clinically and ethically justified.

## Supporting information

S1 Fig When is a PIVC clinically indicated? (PDF)

S2 Fig The lifecycle of a PIVC. (PDF)
