# Supplementary material for: Derivation of a clinical decision-making aid to improve the insertion of clinically indicated peripheral intravenous catheters and promote vessel health preservation. An observational study
Source: PLoS One. 2019 Mar 22;14(3):e0213923. doi: 10.1371/journal.pone.0213923 (PMC6430401; doi:10.1371/journal.pone.0213923)

Appropriate Peripheral IntraVenous Catheter (PIVC) selection with First Time Insertion Sucess (FTIS) and prevention of Premature Device Failure (PDF)

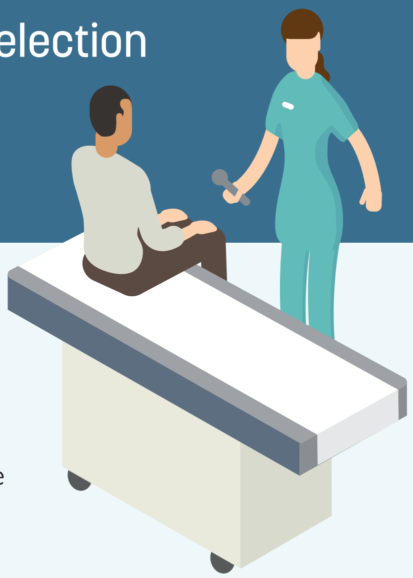

1. Is a PIVC indicated?

- Is an existing device present that can be safely accessed and is appropriate for the following: prescribed intravenous treatment; planned expectant procedures; and/or clinical presentation? If Yes: The indication for a PIVC is not clinically justified.
- Are they likely to require a PIVC for a clinical procedure: Contrast CT Scan; Procedural Sedation; Procedural Access ? If Yes: The indication for a PIVC is clinically justified.
- There are prescribed intravenous fluids that cannot be given enteral route and are appropriate to dilute within peripheral veins. If Yes: The indication for a PIVC is clinically justified.
- The prescribed intravenous medicines cannot be given via the enteral route and are appropriate to dilute within peripheral veins? If Yes: The indication for a PIVC is clinically justified.
- The clinicians treating the patients are concerned that clinical deterioration is likely the insertion risks are outweighed by potential benefit if an untoward event occurred? A clinical serial blood test is required. If Yes: The indication for a PIVC is clinically justified.

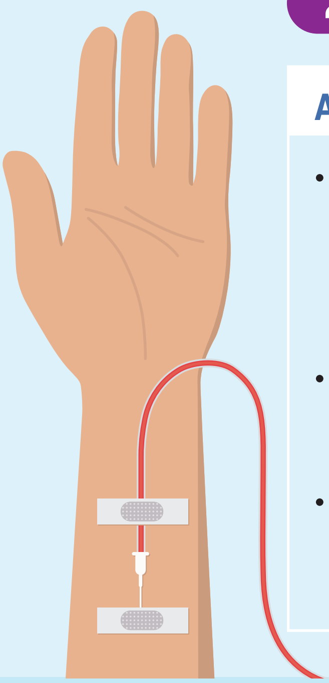

2. A PIVC is indicated

A: Patient Factors

- What patient factors will make this problematic; Can the patient tell you if they are subjected to multiple failed attempts. Is there a record of insertion failure?
- Does the patient represent a demographic that is subjected to PIVC insertion failure (e.g BMI; IVDU; Cancer)?
- What is the vein quality on assessment? Visible and palpable veins suitable for PIVC insertion.

B: Clinician Factors

- What clinician factors influence the success of the procedure?
- Have you informed and shared the decision making process with the patient regarding any potential risks of having a PIVC versus not?
- The clinician role?
- The number of self reported successful insertions?
- The confidence to successfully insert specific to this procedure?

3. Device, Technology and Infusions Factors

- What size peripheral intravenous catheter will you insert? A large one 14-18g or a smaller 20-24g.
- What type of dressing will you use? Is it adhered/ fixed properly?
- Is technology such as ultrasound or near infra-red technology indicated and can the clinician use it?
- Are multiple infusions required?
- Are the infusates damaging to the vein?
- What is the predicted duration of therapy?

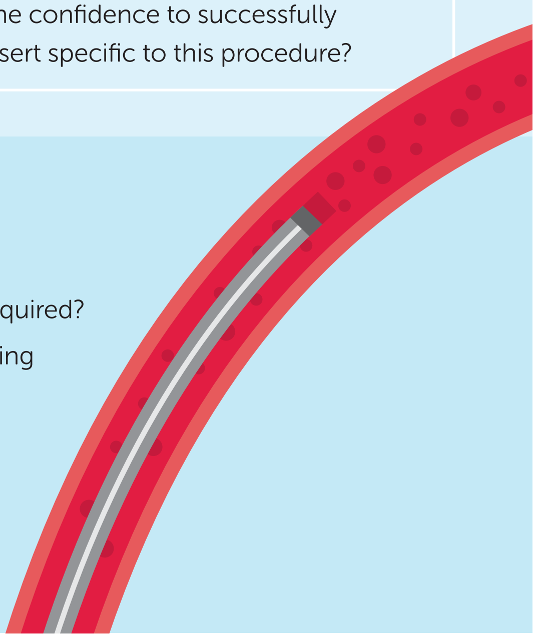

Supplement: S1 Fig — (PDF) [file pone.0213923.s001.pdf]
