# Supplementary material for: Derivation of a clinical decision-making aid to improve the insertion of clinically indicated peripheral intravenous catheters and promote vessel health preservation. An observational study
Source: PLoS One. 2019 Mar 22;14(3):e0213923. doi: 10.1371/journal.pone.0213923 (PMC6430401; doi:10.1371/journal.pone.0213923)
Supplement: S2 Fig — (PDF) [file pone.0213923.s002.pdf]

LEGEND

PIVC Peripheral IntraVenous Catheter
FTIS First Time Insertion Sucess
PDF Premature Device Failure

Successful outcomes intertwine with patient, clinician, products, technology and prescribed infusates.

The cog will turn when a PIVC is clinically indicated.

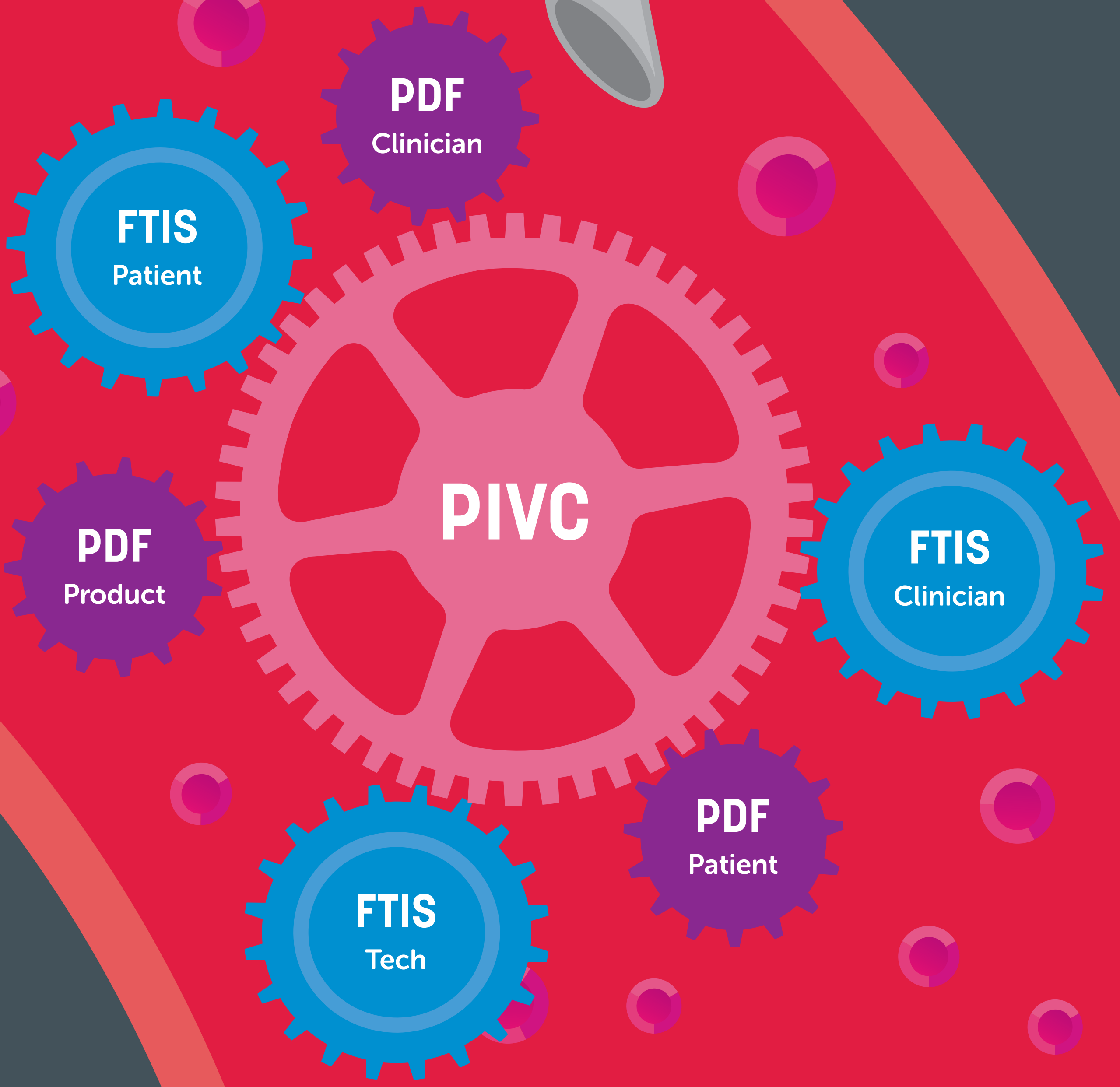

The cog will stop turning when problems occur.
